# Endothelial exosomes contribute to the antitumor response during breast cancer neoadjuvant chemotherapy via microRNA transfer

**DOI:** 10.18632/oncotarget.3520

**Published:** 2015-03-10

**Authors:** Nicolas Bovy, Benoît Blomme, Pierre Frères, Stella Dederen, Olivier Nivelles, Michelle Lion, Oriane Carnet, Joseph A. Martial, Agnès Noël, Marc Thiry, Guy Jérusalem, Claire Josse, Vincent Bours, Sébastien P. Tabruyn, Ingrid Struman

**Affiliations:** ^1^ Laboratory of Molecular Angiogenesis, GIGA-R, University of Liège, Belgium; ^2^ Laboratory of Human Genetics, GIGA-R, University of Liège, Belgium; ^3^ Laboratory of Tumor & Development Biology, GIGA-R, University of Liège, Belgium; ^4^ Laboratory of Cell and Tissues Biology, University of Liège, Belgium; ^5^ Department of Medical Oncology, CHU, Liège, Belgium

**Keywords:** Exosomes, microRNAs, Cancer, miR-503, Angiogenesis

## Abstract

The interaction between tumor cells and their microenvironment is an essential aspect of tumor development. Therefore, understanding how this microenvironment communicates with tumor cells is crucial for the development of new anti-cancer therapies. MicroRNAs (miRNAs) are small non-coding RNAs that inhibit gene expression. They are secreted into the extracellular medium in vesicles called exosomes, which allow communication between cells via the transfer of their cargo. Consequently, we hypothesized that circulating endothelial miRNAs could be transferred to tumor cells and modify their phenotype. Using exogenous miRNA, we demonstrated that endothelial cells can transfer miRNA to tumor cells via exosomes. Using miRNA profiling, we identified miR-503, which exhibited downregulated levels in exosomes released from endothelial cells cultured under tumoral conditions. The modulation of miR-503 in breast cancer cells altered their proliferative and invasive capacities. We then identified two targets of miR-503, CCND2 and CCND3. Moreover, we measured increased plasmatic miR-503 in breast cancer patients after neoadjuvant chemotherapy, which could be partly due to increased miRNA secretion by endothelial cells. Taken together, our data are the first to reveal the involvement of the endothelium in the modulation of tumor development via the secretion of circulating miR-503 in response to chemotherapy treatment.

## INTRODUCTION

The tumor microenvironment includes a variety of cell types: fibroblasts, immune and endothelial cells, pericytes, and local and bone marrow-derived cells, surrounded by matrix components [1]. The crosstalk of this microenvironment with tumor cells is essential for tumor development, adaptation and metastasis formation [2]. Moreover, the blood supply plays a crucial role in cancer progression, allowing access to oxygen and nutrients that support tumor growth and eliminate metabolic waste. Thus, angiogenesis, the process by which new blood vessels arise from preexisting ones, is a crucial step in the progression of tumor development and metastases dissemination, and the blockade of angiogenesis is a promising strategy for the development of new cancer therapies [3]. However, drugs that inhibit tumor blood vessel formation by blocking the VEGF pathway have presently produced limited improvement in the clinical setting [4]. Consequently, a better understanding of the interactions between cancer cells and the tumor microenvironment is necessary to unravel the complexity of tumor physiology and to limit the development of resistance mechanisms during anti-cancer treatments.

MicroRNAs (miRNAs) are small non-coding RNAs that are essential for the regulation of various physiological and pathological processes, including development, differentiation, proliferation and cancer [5, 6]. These transcripts bind to the 3′ untranslated regions (UTRs) of messenger RNAs to either induce their degradation or inhibit their translation into proteins [7,8]. Cell-free miRNAs were found inside exosomes within biological fluids a few years ago [9, 10]. Exosomes are small vesicles, ranging from 30 to 100 nm in size, composed of RNAs, microRNAs, and soluble and membranous proteins [11, 12]. There is accumulating evidence that these organelles play a key role in intercellular communication via the transfer of their molecular contents [13-15]. Furthermore, recent findings demonstrate that circulating miRNAs are promising biomarkers for the diagnosis of several pathologies, demonstrating the notable abilities to discriminate between cancer types and stages and to monitor treatment responses [16-18].

Several studies have demonstrated that tumor exosomes are able to modulate the tumor microenvironment by activating angiogenesis, promoting the formation of cancer-associated fibroblasts (CAFs) and modulating the immune response [19-22]. However, little information is known regarding the role of exosomes derived from cells of the tumor microenvironment on the regulation of tumor cell metabolism. In the present study, we examined the potential transfer of miRNAs from endothelial to tumor cells via exosomes and their role in tumor behavior. We identified the endothelial miRNA miR-503, the expression of which is regulated by breast cancer neoadjuvant chemotherapy and which is able to inhibit tumor cell proliferation and invasion.

## RESULTS

### Endothelial exosomes allow the transfer of miRNAs to tumor cells

**Figure 1:**
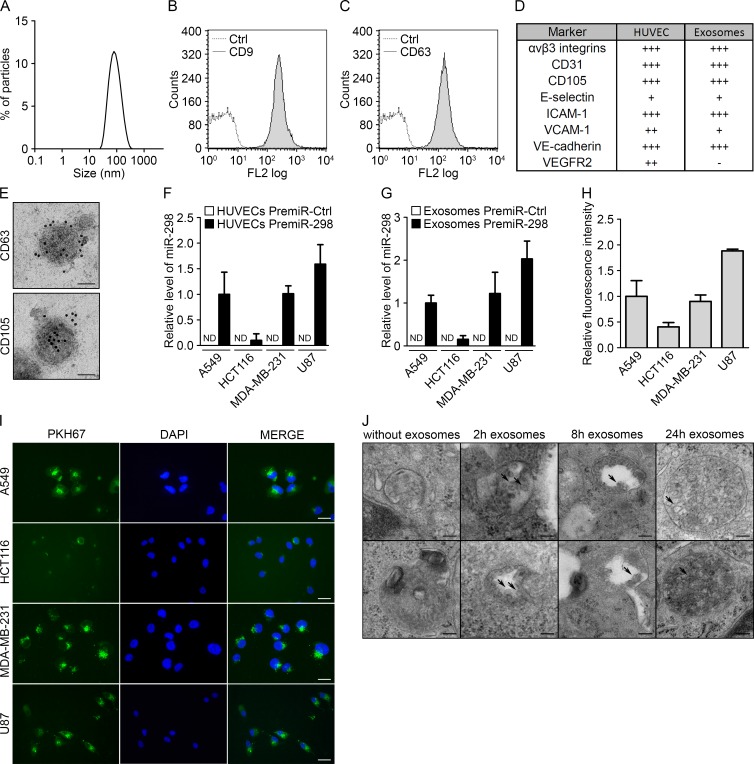
Endothelial exosomes can transfer miRNAs to tumor cells (A) Dynamic light scattering analysis of HUVEC exosomes (max = 94.93). Flow cytometry analysis of HUVEC exosomes immunolabeled for (B) CD9 and (C) CD63. (D) Table summarizing the levels of endothelial markers in HUVECs and HUVEC exosomes, measured using flow cytometry. (E) Electron micrographs of HUVEC exosomes labeled with CD63 and CD105, scale bars = 50 nm. (F) MiR-298 levels evaluated using qRT-PCR in cocultures either of tumor cells with HUVECs transfected with pre-miR-control or pre-miR-298 or (G) of tumor cells incubated with exosomes from HUVECs transfected with pre-miR-control or pre-miR-298. (H) Flow cytometry analysis of the uptake of exosomes (labeled with the green fluorescent PHK67 membrane linker) by tumor cells. (I) Fluorescence microscopy detection of the uptake of PHK67-labeled exosomes by tumor cells (DAPI, blue), scale bars = 25 μm. (J) Electron micrographs of MDA-MB-231 cell sections showing vesicles (arrows); after incubation with HUVEC exosomes for 0, 2, 8 and 24 hours, MDA-MB-231 cells showed larger multivesicular vesicles containing exosomes, scale bars = 100 nm. All data are the mean ± SD (n ≥ 3). *P < 0.05, **P < 0.01 and ***P < 0.001 *vs.* the respective control. Additionally, see Fig. S1.

To investigate the role of circulating endothelial miRNAs on tumor cell physiology, we prospectively isolated and characterized exosomes from human umbilical vein endothelial cells (HUVECs). As demonstrated by dynamic light scattering, exosomes from endothelial cells show the typical size range of these vesicles, with a maximum peak at approximately 95 nm (Fig. 1A). Flow cytometry analysis also confirmed the presence of two well-known exosomal markers, CD63 and CD9, on the exosome surface (Fig. 1B-C). Because the membranous protein composition of the exosomes is representative of the originating cells, we compared the presence of endothelial markers on HUVECs and HUVEC exosomes. As expected, both compartments presented a similar composition of markers, including αvβ3 integrins, CD31, CD105, E-selectin, ICAM-1, VCAM-1 and VE-cadherin. However, VEGFR2, which was strongly expressed on HUVECs, was not found on exosomes (Fig. 1D and Fig. S1A-P). In addition, electron microscopy visualization of the exosomes also revealed a characteristic cup-shaped morphology, with a diameter of approximately 100 nm. Furthermore, immunogold labeling was positive for the exosome marker CD63 and the endothelial marker CD105 (Fig. 1E).

Next, using an exogenous mouse miRNA that is not conserved in humans, mmu-miR-298, we sought to investigate the ability of endothelial cells to transfer miRNAs to human tumor cells. The miRNA was overexpressed in HUVECs, and the transfection efficiency was monitored using qRT-PCR (Fig. S1Q). Transfected HUVECs were then placed in a transwell coculture system with the cells separated by a membrane with 0.2-μm pores to prevent the transfer of miRNAs from other vesicles. This assay was applied to four tumor cell lines (lung carcinoma: A549, colorectal carcinoma: HCT116, breast adenocarcinoma: MDA-MB-231, and glioblastoma: U87) (Fig. 1F). Whereas HCT116 cells presented markedly low levels of mmu-miR-298, the three other tumor cell lines showed significant incorporation of the exogenous miRNA after 48 h. Exosomes were also purified from endothelial cells overexpressing mmu-miR-298, and the presence of the miRNA in exosomes was assessed using qRT-PCR (Fig. S1R). In addition, mmu-miR-298- and control-loaded exosomes were incubated with the various tumor cell lines. As observed in the coculture system, mmu-miR-298 was detected in all cell lines, but HCT116 cells still displayed reduced transfer levels (Fig. 1G).

To study the interaction of endothelial exosomes with tumor cells, we labeled exosomes with the fluorescent lipid dye PKH67 and monitored uptake by the four tumor cell lines. Fluorescence microscopy revealed that all of the cell lines took up the exosomes, but the uptake by HCT116 cells was less pronounced (Fig. 1I). This observation was confirmed via flow cytometry (Fig. 1H). Notably, the exosome incorporation profile was similar to the mmu-miR-298 levels transferred via either coculture or endothelial exosomes, suggesting a major contribution by exosomes in the transfer of miRNAs. Moreover, the variation in uptake efficiencies between different tumor cell types strongly suggests the selective incorporation of endothelial exosomes. To further visualize the mechanism of exosome capture, we monitored exosome uptake over time using electron microscopy. For that experiment, we chose the MDA-MB-231 cell line, as these cells displayed a high level of exosome incorporation. If no exosomes were added to tumor cells, no specific patterns could be observed inside the endocytic vesicles. However, after 2 hours, entities with the characteristic cup shape of exosomes could already be observed inside the endosomes; these entities accumulated over time, as observed after 8 and 24 h (Fig. 1J). These data demonstrate that endothelial exosomes are taken up by tumor cells via endocytosis to allow the intercellular transfer of miRNAs.

### The tumor environment modifies the export of a subset of endothelial miRNAs

**Figure 2:**
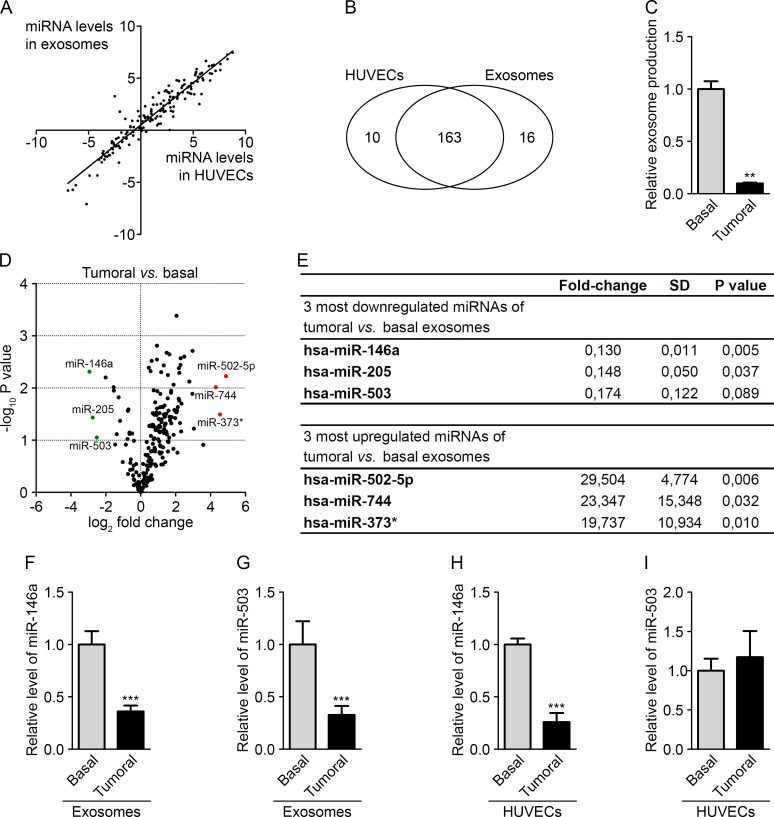
The tumor environment modifies the export of a subset of endothelial miRNAs (A) Dot plot of miRNA levels in HUVECs compared with HUVEC exosomes. (B) Diagram of miRNAs common and specific to HUVECs and HUVEC exosomes. (C) Exosome levels measured by protein quantification from the conditioned medium of HUVECs cultured in basal and tumor-mimicking medium conditions. (D) Volcano plot of fold changes (log_2_ values) and probability values (−log_10_) for individual miRNAs in the exosomes from HUVECs cultured under basal and tumoral conditions. (E) Table of the three most upregulated and downregulated miRNAs under tumoral *vs.* basal conditions. MiR-146a (F) and miR-503 (G) levels, evaluated by qRT-PCR in HUVEC exosomes cultured under tumoral or basal conditions. MiR-146a (H) and miR-503 (I) levels, evaluated by qRT-PCR in HUVECs cultured in tumoral or basal conditions. All data are the mean ± SD (A-E, n = 2; F-I, n = 3). *P < 0.05, **P < 0.01 and ***P < 0.001 *vs.* the respective control. Additionally, see Fig. S2.

Several studies have shown that miRNAs can be transferred from tumor cells to modulate angiogenesis. Here, we speculated that the exchange could also occur in the opposite direction. We hypothesized that tumor cells might elicit an anti-tumor response through the secretion of miRNAs from the endothelium. We thus investigated the miRNA content of endothelial exosomes to identify miRNAs that could modify tumor growth. We first performed miRNA expression profiles using PCR panels (Exiqon) to compare between HUVECs and their exosomes. As observed in other studies [9, 23], most of the miRNAs were expressed at similar levels in cells and exosomes, although some were detected only in cells (10 miRNAs) or in exosomes (16 miRNAs) (Fig. 2A-B and Fig. S2A). To identify endothelial miRNAs that could affect tumor development, we then profiled the miRNA content of exosomes from HUVECs cultured in a basal medium or in a tumor-mimicking medium enriched with growth factors. Basal medium was composed of 5% serum whereas tumoral medium contained a mix of growth factors optimized for HUVECs culture supplemented everyday with high doses of VEGF (50 ng/ml) and bFGF (20 ng/ml). Indeed, these two molecules are well-known activators of tumor angiogenesis [24]. As measured by protein quantification, the first notable observation was the radical decrease in the level of exosome secretion in HUVECs cultured in the tumor medium compared with those cultured in the basal medium (Fig. 2C). Only miRNAs that were detected in all samples, displayed a variation lower than 2 between replicates and an individual Ct value lower than 40 were considered for further analysis. These criteria led to the selection of 204 miRNAs (Fig. S2B). When comparing the miRNA ratio derived from exosomes and HUVECs, 108 miRNAs were found to be modulated in HUVEC-derived exosomes by at least twofold (Fig. 2 D-E and Table S1). As shown in the volcano plot, the 3 most upregulated miRNAs were miR-502-5p, miR-744 and miR-373*, whereas the most downregulated miRNAs were miR-146a, miR-205 and miR-503 (Fig. 2D-E). For further investigation, we examined the 3 most downregulated miRNAs, which, according to our hypothesis, might exhibit antitumor properties. The decreased miR-146a and miR-503 levels in tumoral and basal exosomes were confirmed using TaqMan microRNA assays; however, we were unable to confirm the alteration in miR-205 levels (Fig. 2F-G). Interestingly, we observed that miR-146a levels were also decreased in the exosome-producing endothelial cells, whereas miR-503 levels were not modified under tumor-mimicking conditions (Fig. 2H-I). We then decided to further study miR-503 because this miRNA undergoes a selective export mechanism under tumor-mimicking conditions. In addition, miR-503 is a member of the extended miR-16 family, which has been widely described in the literature as an anti-tumor miRNA that regulates cell cycle progression and the proliferation status of cells [25-27].

### Endothelial miR-503 impairs tumor growth *in vitro*

**Figure 3:**
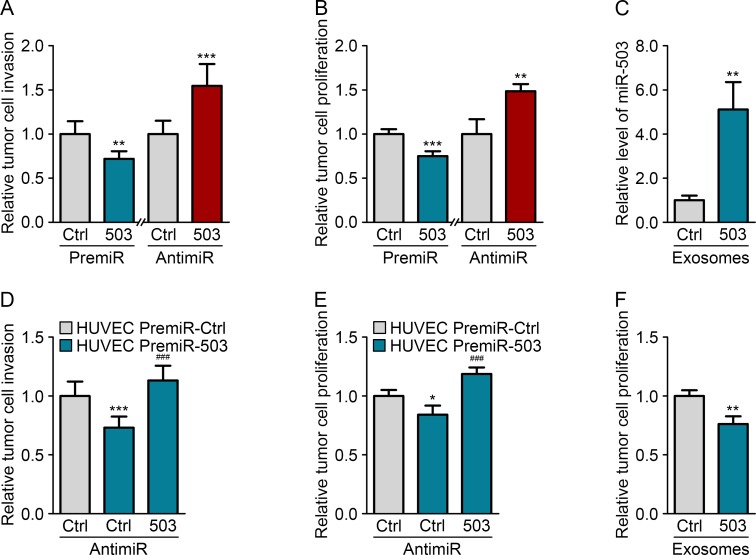
Endothelial miR-503 impairs tumor growth *in vitro* (A) Invasion level of MDA-MB-231 cells transfected with pre-miR-control or pre-miR-503 and with anti-miR-control or anti-miR-503. (B) Luminescence quantification of MDA-MB-231 cells transfected with pre-miR-control or pre-miR-503 and with anti-miR-control or anti-miR-503. (C) MiR-503 levels, measured by qRT-PCR in MDA-MB-231 cells incubated with 5 μg of HUVEC exosomes for 24 h. (D) Invasion level of MDA-MB-231 cells transfected with anti-miR-control or anti-miR-503 and cocultured with HUVECs transfected with pre-miR-control or pre-miR-503. (E) Luminescence quantification of MDA-MB-231 cells transfected with anti-miR-control or anti-miR-503 and cocultured with HUVECs transfected with pre-miR-control or pre-miR-503. (F) Luminescence quantification of MDA-MB-231 cells incubated with exosomes from HUVECs transfected with pre-miR-control or pre-miR-503. Additionally, see Fig. S3.

To investigate the impact of miR-503 on tumor growth, we performed gain- and loss-of-function studies. MDA-MB-231 cells were transfected with either pre- or anti-miR-503, and the transfection efficiency was monitored using qRT-PCR (Fig. S3A). We used MDA-MB-231 constitutively expressing luciferase to quantify the proliferation in a coculture system by measuring the luminescence. Moreover, tumor cell invasion was assessed by quantifying the sprouting of tumor spheroids in a 3D collagen matrix. Increasing miR-503 levels via the transfection of miRNA mimics (pre-miRs) in MDA-MB-231 cells decreased both cell proliferation and invasion. Conversely, inhibition of miR-503 via the transfection of miR-503 antisense LNAs (anti-miRs) resulted in increased levels both of these processes compared with those of the control (Fig. 3A-B). Moreover, the effects of modulating miR-503 on tumor proliferation and invasion were confirmed by measuring BrdU incorporation and performing Boyden chamber assays, respectively (Fig. S3B-C).

We next sought to explore the effect of endothelial-derived miR-503 on MDA-MB-231 cells. For this purpose, HUVECs were transfected with pre-miR-503, and the transfection efficiency was monitored using qRT-PCR (Fig. S3D). The effective transfer of the miRNA was then assessed using endothelial exosomes loaded with miR-503. Upon incubation of miR-503-loaded exosomes with MDA-MB-231 cells, we observed increased miRNA levels in the cells using qRT-PCR (Fig. 3C). We next investigated whether miR-503 secreted from endothelial cells could modify the phenotype of MDA-MB-231 cells. HUVECs overexpressing miR-503 were cocultured with MDA-MB-231 cells, which led to reduced tumor cell proliferation and invasion compared with those under control conditions (Fig. 3D-E). Importantly, the addition of anti-miR-503 into tumor cells rescued the effects of endothelial miR-503 in both functional assays. This observation suggest that miR-503 is the main effector of tumor cell proliferation and invasion inhibition in endothelial exosomes. To confirm that these effects were caused by the transfer of endothelial miR-503 via exosomes, we treated MDA-MB-231 cells with miRNA-loaded HUVEC exosomes. This treatment also resulted in reduced MDA-MB-231 proliferation compared with the cells treated with mock HUVEC exosomes (Fig. 3F).

### MiR-503 inhibits CCND2 and CCND3 expression of MDA-MB-231

**Figure 4:**
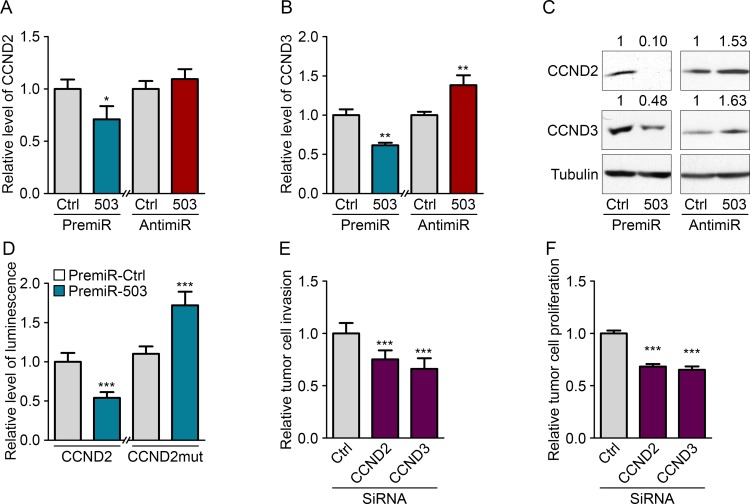
MiR-503 inhibits CCND2 and CCND3 expression of MDA-MB-231 Quantification of mRNA levels according to qRT-PCR of (A) CCND2 and (B) CCND3 in MDA-MB-231 cells transfected with pre-miR-503 or pre-miR-control and with anti-miR-control or anti-miR-503 and starved for 36 h. (C) Western blotting of CCND2 and CCND3 in MDA-MB-231 cells transfected with pre-miR-503 or pre-miR-control and with anti-miR-control or anti-miR-503 and starved for 36 h. (D) Luciferase activity from the CCND2 3′-UTR WT reporter plasmid and mutated NRAS 3′-UTR reporter plasmid cotransfected into MDA-MB-231 cells with pre-miR-control or pre-miR-503 after 24 hours. (E) Invasion levels of MDA-MB-231 cells transfected with siRNAs targeting CCND2 and CCND3. (F) Luminescence quantification of MDA-MB-231 cells transfected with siRNAs targeting CCND2 and CCND3. *P < 0.05, **P < 0.01 and ***P < 0.001 *vs.* the respective control. ^###^P < 0.001 *vs.* miR-503-exosomes with anti-miR-control. Additionally, see Fig. S4.

To explore the molecular mechanism responsible for the inhibition of tumor cell proliferation and invasion by miR-503, we searched for target genes of the miRNA involved in those mechanisms. We used a computational approach involving the Targetscan algorithm (http://www.targetscan.org/) to obtain a list of genes predicted to be targets of miR-503. We then used the STRING algorithm (http://string-db.org/), which creates a network between proteins that have functional or physical interactions, to identify associations between predicted targets of miR-503. We observed the presence of a large cluster of proteins that influence cell cycle progression and pathways that regulate the proliferation/apoptosis status of cells (Fig. S4C). Using qRT-PCR, we tested a subset of miR-503 target genes that displayed many links with each other to identify the genes responsible for the observed anti-tumor phenotype. From this subset of genes, we identified two targets of miR-503, CCND2 and CCND3, which are downregulated at the RNA and protein levels upon overexpression of the miRNA (Fig. 4A-C). Importantly, inhibiting endogenous miR-503 using anti-miR transfection led to increased CCND2 and CCND3 protein levels. The interaction sites of miR-503 and the CCND2 and CCND3 3′-UTRs are pictured in Fig S4D. Notably, expression of the homologue CCND1 was not affected by miR-503 even though this gene is a validated target of miR-503 (Fig. S4A-B). Because CCND2 has never been described as a target of miR-503, we analyzed whether the miRNA directly interacts with its 3′-UTR. Indeed, there are 3 binding sites for miR-503 in the CCND2 3′-UTR: one 8-mer site and two 7-mer-1A sites. We therefore constructed a luciferase reporter vector that encoded the 3′-UTR of CCND2 downstream of the luciferase coding sequence. Reduced luciferase activity was observed in MDA-MB-231 cells transfected with the vector; these cells were also observed to overexpress miR-503 compared with that of the control (Fig. 4D). Moreover, when the sequences that bind the seed region of miR-503 were mutated in the CCND2 3′-UTR, the miRNA was no longer able to inhibit translation of the luciferase mRNA, leading to normalized luminescence levels.

We next determined whether the inhibition of CCND2 and CCND3 was responsible for the inhibition of tumor cell proliferation and invasion observed upon miR-503 modulation. Indeed, silencing CCND2 and CCND3 in MDA-MB-231 cells with siRNA led to decreased proliferation and invasion (Fig. 4E-F). Taken together, these results demonstrate that endothelial-derived miR-503 induces the inhibition of tumor cell proliferation and invasion via inhibition of CCND2 and CCND3.

### Neoadjuvant chemotherapy increases circulating miR-503 levels

To study the potential endothelial release of miR-503 during cancer, we analyzed the plasmatic miR-503 levels in breast cancer patients subjected to various therapies. Interestingly, we observed increased miR-503 levels in patients receiving neoadjuvant chemotherapy treatment, whereas no changes were observed in patients treated only with surgery (Fig. 4A-B and D-E). To more deeply investigate the influence of neoadjuvant chemotherapy, miR-503 levels were analyzed in tumor biopsies and in residual tumors of patients subjected to this treatment and presenting an incomplete pathological response. Surprisingly, no changes in the miR-503 levels were observed before treatment or after post-chemotherapy surgery (Fig. 4C and F). This observation favors the view that the increased miR-503 levels in the circulation after chemotherapy do not come from the tumor. Thus, we decided to investigate whether endothelial cells could be responsible for the increased miR-503 levels upon neoadjuvant chemotherapy. We treated endothelial cells with both chemotherapeutic agents (epirubicin and paclitaxel) used in the treatment and analyzed the consequences on miR-503 expression and exosome secretion using qRT-PCR. We first observed a drastic increase in exosome production in endothelial cells treated with the chemotherapeutic agents; this effect was more pronounced with paclitaxel than with epirubicin treatment (Fig. 4G). Moreover, we observed increased miR-503 levels in exosomes following epirubicin and paclitaxel treatments compared with control conditions, whereas decreased levels were observed in exosome-producing HUVECs (Fig. 4H-I). These data suggest that the elevated circulating miR-503 levels observed in patients after neoadjuvant chemotherapy could originate in part from exosome modification and miR-503 secretion by endothelial cells.

## DISCUSSION

Over the past few years, exosomes have emerged as important players in intercellular communication. Notably, several studies have demonstrated the role of tumor exosomes in regulating major processes of tumor progression, such as angiogenesis, immune modulation and metastasis dissemination. However, until now, the effects of exosomes secreted from endothelial cells on tumor cells has not been explored. Endothelial exosomes have been shown to induce several mechanisms, such as the regulation of angiogenesis in other endothelial cells [28, 29] and the atheroprotective stimulation of smooth muscle cells [15]. It is well known that cells within the tumor microenvironment can act on tumor cells. In this context, a role for exosomes has emerged in the recent literature. For example, exosomes secreted from mesenchymal stem cells can regulate tumor growth, whereas exosomes from dendritic cells can induce tumor regression [30-32]. In this study, we demonstrate that endothelial cells, which are also important players in the tumor environment, produce exosomes that are able to transfer miRNAs to tumor cells. Our data reveal that this transfer involves endocytosis because incorporated exosomes are observed in endocytic vesicles inside tumor cells.

An important finding of our work is the observation that the miRNA content of endothelial exosomes differs from that of the producing cells. This phenomenon has been described in other studies and demonstrates that, at least in part, miRNAs can be selectively packaged into exosomes [9, 23]. In addition, our profiling experiment also revealed that the miRNA content of exosomes is altered by the culture conditions. This experiment led to the identification of miR-503, the exosome levels of which are decreased under tumor conditions despite cellular levels being unaffected. Notably, miR-424, which belongs to the same cluster as miR-503, also showed reduced levels under the same conditions. MiR-503 is known to inhibit proliferation, migration and tube formation in endothelial cells under high-glucose stress conditions [33]. Moreover, miR-503 has been widely described in the literature as an anti-tumor miRNA that regulates the expression of key cell cycle proteins, such as CDC25A and cyclins D1 and E1, as well as cell proliferation via E2F3 and PI3K regulation and the apoptosis status via BCL-2 inhibition [6, 25-27, 33, 34]. Notably, miR-503 also prevents tumor growth by interacting with the tumor microenvironment and reducing secretion of the proangiogenic factors FGF2 and VEGF by tumor cells [35]. We obtained similar results by modulating the expression of miRNA in MDA-MB-231 cells. The overexpression of miR-503 led to decreased tumor cell proliferation and invasion in multiple assays, whereas its inhibition presented opposite results. The role of endothelial miR-503 contained in exosomes has also been investigated by coculturing HUVECs overexpressing miR-503 with tumor cells. This manipulation also led to reduced proliferative and invasive properties, which could be reversed by adding anti-miR-503 to MDA-MB-231 cells. During investigation of the mRNA targets of miR-503 to explain these effects, we identified that miR-503 regulated cyclins D2 and D3. Cyclin D3 is already a validated target of miR-503; however, the regulation of the cyclin D2 had not yet been demonstrated [26]. Overall, these data showed that endothelial cells cultured under tumoral conditions released miR-503, which can have antitumoral effects, into the medium.

**Figure 5:**
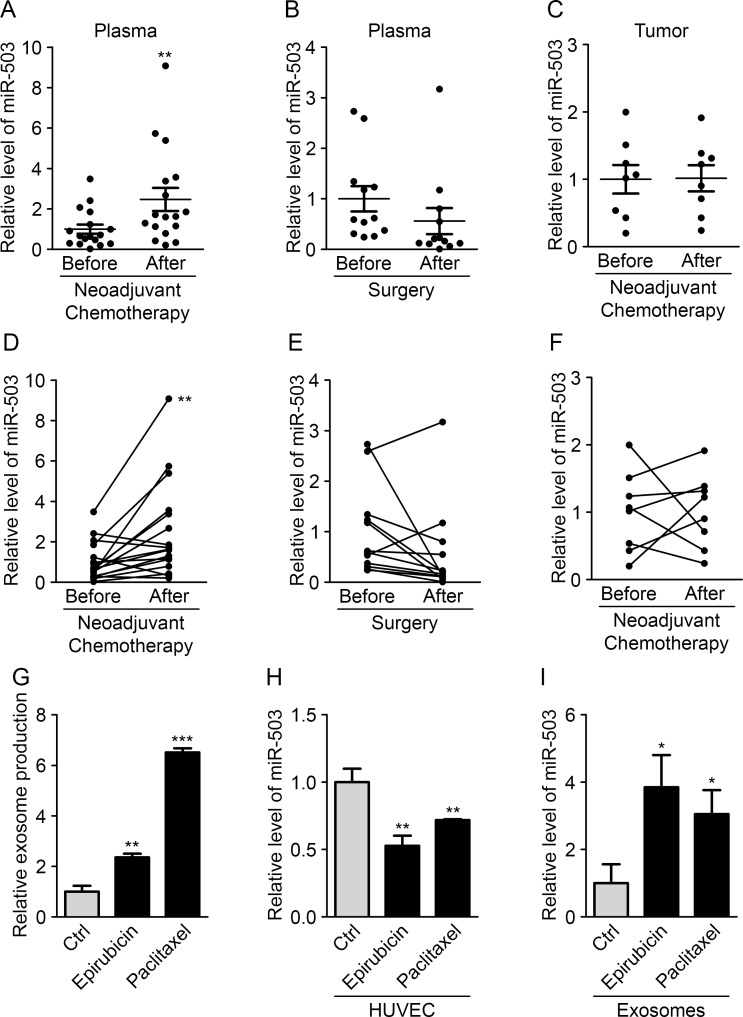
Neoadjuvant chemotherapy increases circulating miR-503 levels (A) The miR-503 ratio according to qRT-PCR in blood samples of breast cancer patients before and after neoadjuvant chemotherapy. (B) The miR-503 ratio according to qRT-PCR in blood samples of breast cancer patients before and after surgery. (C) The miR-503 ratio according to qRT-PCR in tumor biopsies and residual tumors of breast cancer patients. (D) Individual follow-up of miR-503 levels, evaluated by qRT-PCR, in blood samples of breast cancer patients before and after neoadjuvant chemotherapy. (E) Individual follow-up of miR-503 levels, evaluated by qRT-PCR, in blood samples of breast cancer patients before and after surgery. (F) Individual follow-up of miR-503 levels, evaluated by qRT-PCR, in tumor biopsies and residual tumors of breast cancer patients. (G) Exosome levels, measured by protein quantification, from the conditioned medium of HUVECs treated with paclitaxel and epirubicin for 24 h. (H) The miR-503 levels, evaluated by qRT-PCR, in HUVECs treated with paclitaxel and epirubicin for 24 h. (I) The miR-503 levels, evaluated by qRT-PCR, in exosomes from HUVECs treated with paclitaxel and epirubicin for 24 h. *P < 0.05, **P < 0.01 and ***P < 0.001 *vs.* the respective control.

Our human studies revealed a role for miR-503 in response to neoadjuvant therapy. Plasmatic miR-503 levels were elevated in breast cancer patients receiving neoadjuvant chemotherapy. As suggested by our *in vitro* data, the elevation of miR-503 in the blood after chemotherapy could originate, at least in part, from the increased secretion of miR-503 by endothelial cells following paclitaxel and epirubicin treatment. Because decreased miR-503 expression was observed in exosome-producing endothelial cells, it is likely that the chemotherapeutic agent promotes the transfer of miR-503 to exosomes rather than the induction of its expression. A similar effect has been reported in endothelial cells submitted to ionizing radiation [36]. On the other hand, plasmatic levels of miR-503 of patients treated only with surgery, and tumor levels of miR-503 of patients under chemotherapy were not affected. Therefore, it is likely that endothelial circulating miR-503 would originate from the entire endothelium. As previously described by other studies, anthracyclines and taxanes induce an endothelial toxicity which also affects endothelial cells outside of the tumor [37]. Therefore, miR-503 might act as a stress-induced miRNA that is essential for cell cycle regulation. Its expression is increased upon serum starvation of mesenchymal stem cells and is modulated according to cell cycle progression [38, 39]. Thus, we propose a model in which endothelial cells, in response to unfavorable conditions, such as chemotherapy or radiation treatment, release circulating miR-503 into the surrounding environment (Fig. 5). Endothelial exosomes loaded with miR-503 might then inhibit tumor growth by acting directly on tumor cells, thereby contributing to the direct effect of these therapies. To the best of our knowledge, this is the first report of a miRNA transferred from endothelial cells to tumor cells via exosomes. Our data also reveal the involvement of the endothelium in the modulation of tumor development upon chemotherapy treatment. In this context, miR-503 appears to be an antitumor miRNA secreted by endothelial cells that is able to regulate tumor cell proliferation and invasion via the inhibition of CCND2 and CCND3. This process might complement the direct effects of chemotherapy and thereby help the host fight the tumor.

**Figure 6:**
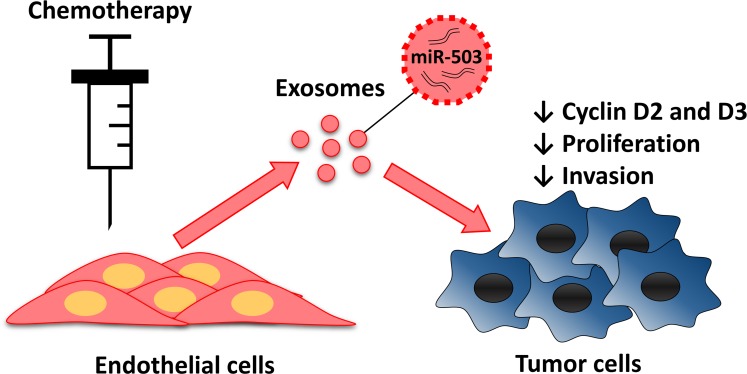
Endothelial transfer of miR-503 elicit antitumor response during neoadjuvant chemotherapy Endothelial cells treated with chemotherapeutic agents release more exosomes that contain more miR-503. MiR-503 loaded exosomes induce a reduction of breast cancer cells proliferation and invasion caused by the inhibition of CCND2 and CCND3.

## MATERIALS AND METHODS

### Cell culture

The isolation and culture of HUVECs (passages 6–11) were previously described [40]. A549 and U87 cells were cultured in EMEM supplemented with 10% FBS. HCT116 cells were cultured in McCoy's 5A medium supplemented with 10% FBS. MDA-MB-231 cells were cultured in DMEM 4500 supplemented with 10% FBS.

### Cell transfections and treatments

Pre-miRs (25 nM; Ambion) and anti-miRs (25 nM; Exiqon) were transfected into HUVECs and MDA-MB-231 cells using Dharmafect-4 (Dharmacon Research Inc.) according to the manufacturer's instructions. CCND2, CCND3 and control siRNAs (20 nM) were transfected based on the calcium phosphate transfection method. Transfected HUVECs and MDA-MB-231 cells were plated in EGM2 or complete DMEM, respectively. After a 24-hour transfection, the cells were washed and kept in their media for an additional 48 or 72 hours. Functional assays were performed as described above.

### Exosome purification

Exosomes were isolated and purified from the supernatants of HUVEC cultures using the differential centrifugations. HUVECs were cultured in EGM2 medium containing exosome-depleted serum. After 72 h, the medium was collected and centrifuged at 2000 g for 15 min at 4°C and then again at 12,000 g for 45 min at 4°C. Supernatants were then passed through a 0.22-μm filter (Millipore) and ultracentrifuged at 110,000 g for 90 min at 4°C. The pellets were then washed with phosphate buffer saline (PBS), followed by a second ultracentrifugation at 110,000 g for 90 min at 4 °C, then resuspended in PBS. The protein levels of the exosome preparations were measured using the BCA Protein Assay kit (Pierce) following the manufacturer's instructions.

### PKH67 labeling of exosomes

Exosomes were labeled with PKH67 dye (Sigma) according to the manufacturer's instructions, and incubated with cells for 24 h. The cells were then washed 2 times with PBS and mounted on a slide for observation under a fluorescence microscope.

### Dynamic light scattering

Exosomes were suspended in PBS at a concentration of 50 μg/mL, and analyses were performed with a Zetasizer Nano ZS (Malvern Instruments, Ltd.). Intensity, volume and distribution data for each sample were collected on a continuous basis for 4 min in sets of 3.

### Flow cytometry

Cells were first saturated for 30 min in PBS containing 5% BSA and incubated for 1 h at 4°C with the following antibodies: anti-αvβ3 integrin (Chemicon), anti-CD9 (Santa Cruz), anti-CD31 (Dako), anti-CD63 (BD-Biosciences), anti-CD105 (Dako), anti-E-selectin (Abcam), anti-ICAM-1 (Abcam), anti-V-CAM1 (Abcam), anti-VE-Cadherin (Enzo Life Sciences) and anti-VEGFR2 (Cell Signaling). Cells were then incubated with a biotin-coupled secondary antibody for 30 min and then incubated with streptavidin-PE for 30 min before being analyzed on the FACSCalibur flow cytometer (BD Biosciences). Exosomes were incubated for 30 min with latex beads (Invitrogen), then the exosome-coated beads were subjected to the same steps.

### Patients

Ethical approval was obtained from the Institutional Review Board (Ethical Committee of the Faculty of Medicine of the University of Liège) in compliance with the Declaration of Helsinki. Patients with newly diagnosed primary breast cancer were prospectively recruited at CHU of Liège (Liège, Belgium) from July 2011 to July 2013. All patients signed a written informed consent form. This work consisted of a prospective study and did not influence the treatment of the enrolled patients; 29 patients were included in this study. Blood samples were collected into 9-mL EDTA-containing tubes. Plasma was prepared within 1 h by retaining the supernatant after double centrifugation at 4°C (10 min at 815 g and 10 min at 2500 g), then stored at −80°C.

Seventeen patients with primary locally advanced breast cancer received neoadjuvant chemotherapy (NAC) with 3 or 4 courses of alkylating agents (cyclophosphamide or fluorouracil) and anthracycline-based chemotherapy (epirubicin) followed by 3 or 12 courses of tubulin-binding agents (docetaxel or paclitaxel). Eight of these patients did not achieved pathological complete response (ypT0N0, following the AJCC-UICC classification)

For those 8 non responders, 4-μm tumor slices from formalin-fixed paraffin-embedded (FFPE) tissues samples were obtained from diagnostic core-needle biopsies (2 to 3 passes in the primary tumor) and the corresponding remaining tumor. The histological statuses of all 8 tissues samples were established with hematoxylin and eosin staining of the FFPE sections by a pathologist.

Circulating miR-503 levels were also analyzed in a cohort of twelve primary breast cancer patients that did not receive any chemotherapy, and their plasma was collected 8 days before and 3 months after surgery.

### Electron microscopy of whole-mounted immuno-labelled exosomes

Exosome were placed on Formvar-carbon coated nickel grids for 1 h, washed 3 times with PBS and fixed with 2% paraformaldehyde for 10 min. After 3 washes, grids were then incubated for 2 h with the following antibodies: anti-CD63 or anti-CD105. Exosomes were then washed 5 times and incubated with a 10 nm-gold labeled secondary antibody. They were washed 5 more times and post-fixed with 2.5% glutaraldehyde for 10 min. Samples were contrasted using 2.5% uranyl acetate for 10 min followed by 4 washes and an incubation of 10 min in lead citrate. Grids were finally washed 4 times in deionized water and examined with a JEOL JEM-1400 transmission electron microscope at 80 kV.

### Electron microscopy of exosome uptake in MDA-MB-231

MDA-MB-231 cells were treated with exosomes (final concentration, 20 μg/mL) dissolved in PBS. After 0, 2, 8 and 24 h pre-incubation, the MDA-MB-231 cells were washed with PBS and fixed in 2.5% glutaraldehyde/1% paraformaldehyde in 0.1 M PBS (pH 7.4) for 2 h at 4°C. Then, the cells were washed 3 times in PBS for 10 min at 4°C and post-fixed for 30 min with 2% osmium tetroxide. After dehydration in graded ethanol, samples were embedded in Epon. Ultrathin sections were obtained with a Reichert Ultracut S ultramicrotome then contrasted with uranyl acetate and lead citrate. Observations were made with a JEOL JEM-1400 transmission electron microscope at 80 kV.

### MicroRNA profiling

Total RNA was extracted with the miRNeasy kit (Qiagen) following the manufacturer's protocol, and cel-miR-39 and cel-miR-238 were spiked into the exosome samples. Reverse transcription was then performed using the miRCURY LNA™ Universal RT microRNA PCR, polyadenylation and cDNA synthesis kit (Exiqon, Denmark). Quantitative PCR was performed according to the manufacturer's instructions on microRNA Ready-to-Use PCR panel 1. The controls included reference genes, inter-plate calibrators run in triplicate (Sp3) and negative controls.

### Cell coculture and functional assays

For cocultures, endothelial donor cells were seeded onto 6-well plates. After 8 hours, transwells were added, and tumor cells were seeded onto the inner part of the transwell membranes. After 48 h of incubation, tumor cells were collected and analyzed.

### Proliferation assays

For the luminescence proliferation assay, MDA-MB-231 cells were transfected or incubated with transfected HUVECs on a 24-well plate. After 48 h, 150 μg/mL of luciferin was added per well, and the luminescence was quantified using the bioluminescent IVIS imaging system (Xenogen-Caliper).

For the BrdU incorporation assay, MDA-MB-231 cells were transfected and seeded into 96-well plates for 40 h. BrdU was added for 8 h, and proliferation was assessed using the Cell Proliferation ELISA BrdU (colorimetric) kit (Roche) following the manufacturer's instructions.

### Spheroid invasion assay

Spheroids were prepared as previously described [41]. Briefly, spheroids composed of transfected MDA-MB-231 alone or with transfected HUVECs were allowed to form in 96-U-well suspension plates for 48 h. Spheroids were then collected and seeded for 48 h inside a 3D collagen matrix with culture medium. Pictures were taken to quantify the invasion level by measuring the area of invasion using ImageJ software.

### Boyden chamber assay

Transfected MDA-MB-231 cells were seeded into 8-μm 24-well Boyden chambers (Transwell; Costar Corp) and subjected to cell invasion assays. The lower chamber was filled with 600 μL of complete DMEM, and transfected MDA-MB-231 cells were placed in 300 μL of free DMEM in the upper chamber and allowed to migrate for 4 h at 37°C. After fixation, cells were stained with 4% Giemsa and counted on the lower side of the membrane using ImageJ software.

### Chemotherapy treatment of endothelial cells

HUVECs were cultured in EGM2 supplemented with 1 μg/mL epirubicin or 20 ng/mL paclitaxel. After 24 h, the medium was swapped with exosome-depleted medium, and the cells were cultured for 72 additional hours before being collected for exosome purification.

### Preparation of cell extracts and Western blot analysis

Cells were washed with PBS and scraped into lysis buffer [50 mM Tris-HCl (pH = 7.5); 1% NP40; 0.5% sodium desoxycholate; 1 mM EDTA; protease inhibitor cocktail cOmplete Mini, EDTA free (Roche)] on ice. Insoluble cell debris was removed by centrifugation at 10,000 g for 15 min. Aliquots of protein-containing supernatant were stored at −20°C, and protein concentrations were measured using the BCA Protein Assay kit (Pierce) following the manufacturer's instructions.

Soluble cell lysates (50 μg) were resolved using SDS-PAGE (12%) and transferred to polyvinylidene fluoride membranes (Millipore). Blots were blocked overnight with 8% milk in Tris-buffered saline with 0.1% Tween 20 and probed for 1 h with the following primary antibodies: anti-CCND1 (Cell Signaling), anti-CCND2 (Cell Signaling), anti-CCND3 (Cell Signaling), and anti-beta-tubulin (ab6046, Abcam). After 3 washes with Tris-buffered saline containing 0.1% Tween 20, antigen-antibody complexes were detected with a peroxidase-conjugated secondary antibody and the enhanced fluoro-chemiluminescent system (ECL; Pierce Biotechnology). Quantifications were performed using ImageJ software and are presented in bar graphs normalized to the levels of the corresponding loading control (β-tubulin).

### RNA extraction, miRNA and mRNA expression analysis using the TaqMan microRNA assay and quantitative real-time PCR analysis

Total RNA was extracted using the miRNeasy kit (Qiagen) following manufacturer's protocol, and cel-miR-39 and cel-miR-238 were spiked into exosome and plasma samples.

TaqMan assays were used to assess miRNA expression. Briefly, 10 ng RNA was reverse transcribed into cDNA using the TaqMan microRNA Reverse Transcription kit and the TaqMan microRNA assay stem loop primers (Applied Biosystems). The resulting cDNAs were used for quantitative real-time PCR using the TaqMan microRNA assay and TaqMan universal PCR master mix reagents (Applied Biosystems). Thermal cycling was performed on an Applied Biosystems 7900 HT detection system (Applied Biosystems). For the cells, the relative miRNA levels were normalized to 2 internal controls, RNU-44 or RNU-48. For the plasma and exosomes, the relative miRNA levels were normalized to the 2 spiked-in miRNAs: cel-miR-39 and cel-miR-238 (Applied Biosystems).

For mRNA expression analysis, RNA was extracted using the miRNeasy kit (Qiagen) according to the manufacturer's protocol. cDNA synthesis was performed with 1 μg of total RNA and the iScript cDNA Synthesis kit (BioRad), according to the manufacturer's instructions. The resulting cDNA transcripts (20 ng) served as the template for quantitative real-time PCR using the SYBR green method (Roche Applied Sciences). Thermal cycling was performed on an ABI Prism 7900 HT Sequence Detection System (Applied Biosystems). For all reactions, no-template controls were run, and random RNA preparations were also subjected to sham reverse transcription to verify the absence of genomic DNA amplification. Quantitative real-time PCR was performed using the SYBR green method (Bioline and Thermo Fisher Scientific). Thermal cycling was performed on an Applied Biosystems 7900 HT detection system (Applied Biosystems). The relative transcript level of each gene was normalized to the housekeeping genes cyclophilin-A (PPIA) and/or glyceraldehyde 3-phosphate dehydrogenase (GAPDH). Primers were designed using Primer Express software and selected to span exon-exon junctions to avoid the detection of genomic DNA (primer sequences are provided below).

**Table 1:** Primer sequences

Gene	Forward primer	Reverse primer
hsa CCND1	CAATGACCCCGCACGATTTC	CATGGAGGGCGGATTGGAA
hsa CCND2	CTCGAGGGATGCCAGTTGGGCC	GCGGCCGCCAAAAGCGTGAATCATTGCC
has CCND3	TACCCGCCATCCATGATCG	AGGCAGTCCACTTCAGTGC
has GAPDH	GCATCTTCTTTTGCGTCGC	CCAAATGCGTTGACTCCGA
has PPIA	CCAACACAAATGGTTCCCAGT	CCATGGCCTCCACAATATTCA

name	Sense strand	Antisense strand
siRNA CCND2	GGCAUGAUUAGAUUGCAAAGCAATG	CAUUGCUUUGCAAUCUAAUCAUGCCAU
siRNA CCND3	CUUACUGUAAUAAAGAUGAUUGUGA	UCACAAUCAUCUUUAUUACAGUAGGAU

### Sequences of qRT-PCR primers, siRNAs and antagomir

All primers, siRNAs and control siRNAs were synthesized by IDT-DNA

### Luciferase assay (3′-UTR reporter assays)

MDA-MB-231 cells were transfected with 30 pmol of pre-miR-Ctrl or pre-miR-503 with 0.6 μL of DharmaFECT-4 (Dharmacon Research, Inc.). The next day, the cells were transfected with 0.1 μg of the psiCHECK2 vector (Promega) expressing the 3′-UTR of the human *CCND2* mRNA (nucleotides 1-2040) or the mutated 3′-UTR of the human *CCND2* mRNA (QuickChange II Site-Directed Mutagenesis Kit, Agilent) with JET-PEI (Polyplus transfection) according to the manufacturer's instructions. The wild-type region of the 3′-UTR human *CCND2* mRNA miR-503 binding seed sequences were 1: 5′-GCUGCUA-3′, 2: 5′-CGCUGCUA-3′ and 3: 5′-GCUGCUA-3′. The mutated region of the 3′-UTR human *CCND2* mRNA miR-503 binding seed sequence were 1: 5′-AAUAAUA-3′, 2: 5′-CAAUUUUA-3′ and 3: 5′-AAUAAUA-3′. After 24 h, the luciferase assay was performed using the Dual-Luciferase Reporter Assay System (Promega). Renilla luciferase activity was normalized to firefly luciferase activity.

### Data analysis

All values are expressed as the mean ± SD (*in vitro* experiments) or the mean ± SEM (patient analyses). Comparisons between various conditions were assessed using two-tailed Student's t tests. Analyses of patients before and after treatments (Fig. 4) were performed using two-tailed paired t tests. P values less than 0.05 were considered statistically significant.

## SUPPLEMENTARY MATERIALS, FIGURES AND TABLES


