# Supplementary material for: Endothelial exosomes contribute to the antitumor response during breast cancer neoadjuvant chemotherapy via microRNA transfer
Source: Oncotarget. 2015 Mar 10;6(12):10253–66. doi: 10.18632/oncotarget.3520 (PMC4496353; doi:10.18632/oncotarget.3520)
Supplement: Supplementary file 1 [file oncotarget-06-10253-s001.pdf]

Endothelial exosomes contribute to the antitumor response during breast cancer neoadjuvant chemotherapy via microRNA transfer

Supplementary Material

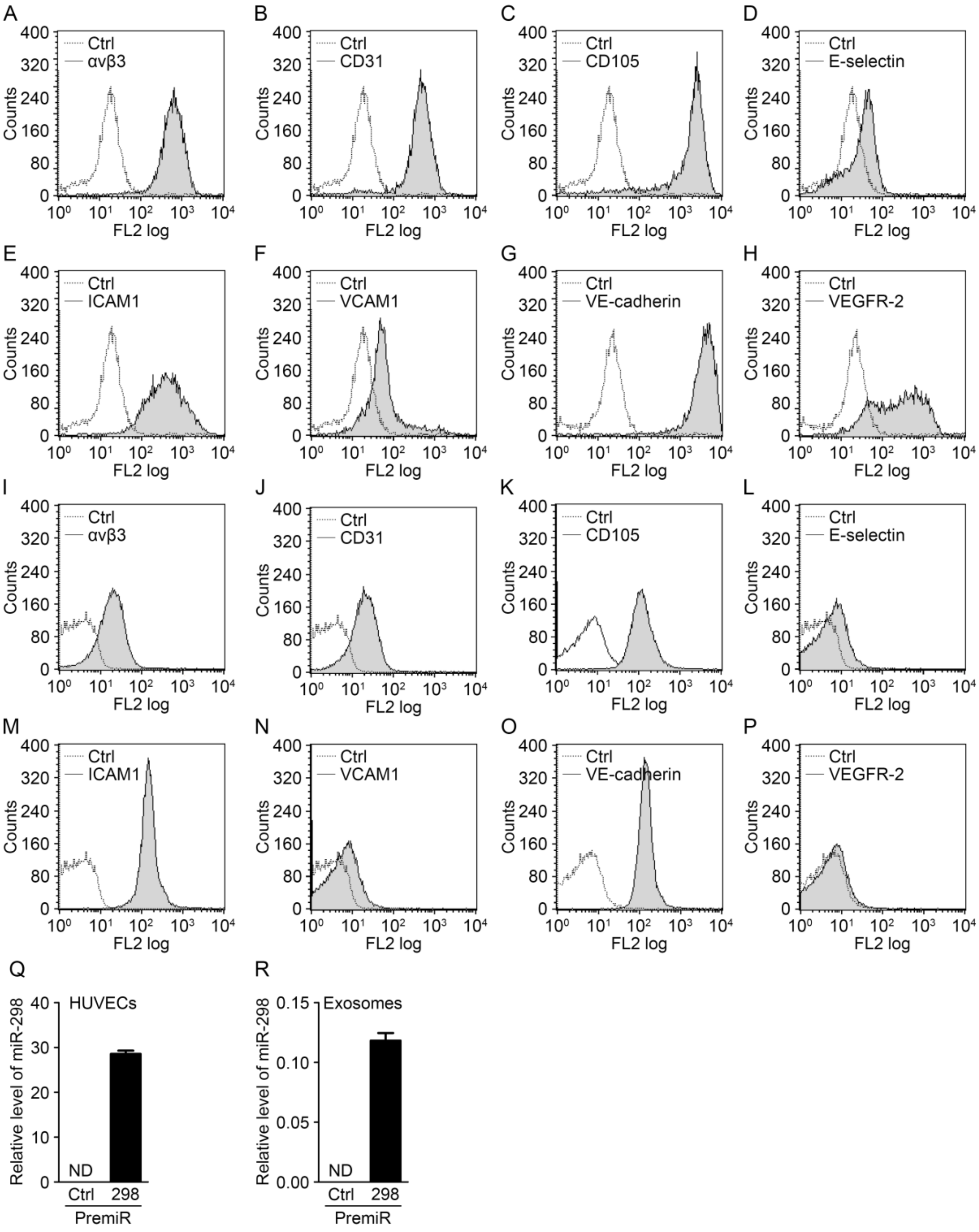

Figure S1

(A-H) Flow cytometry analysis of HUVECs for the levels of (A) αvβ3 integrins, (B) CD31, (C) CD105, (D) E-selectin, (E) ICAM-1, (F) VCAM-1, (G) VE-cadherin and (H) VEGFR2. (I-P) Flow cytometry analysis of HUVEC exosomes for the levels of (I) αvβ3 integrins, (J) CD31, (K) CD105, (L) E-selectin, (M) ICAM-1, (N) VCAM-1, (O) VE-cadherin and (P) VEGFR2. (Q) MiR-298 levels evaluated by qRT-PCR in HUVECs transfected with pre-miR-298 or pre-miR-control. (R) MiR-298 levels evaluated by qRT-PCR in the exosomes of HUVECs transfected with pre-miR-298 or pre-miR-control.

A

miRNAs only detected in exosomes	Common miRNAs between HUVECs and exosomes	miRNAs only detected in HUVECs
miR-122, miR-133a, miR-133b, miR-142-3p, miR-142-5p, miR-143, miR-144, miR-150, miR-202, miR-223, miR-451, miR-486-5p, miR-506, miR-509-3p, miR-596, miR-631	let-7b, let-7d, let-7d*, let-7g, let-7i, miR-101, miR-106a, miR-107, miR-10b, miR-125a-5p, miR-125b, miR-126, miR-126*, miR-128, miR-130a, miR-130b, miR-132, miR-134, miR-136, miR-137, miR-138, miR-139-5p, miR-140-3p, miR-140-5p, miR-146a, miR-148a, miR-148b, miR-149, miR-151-3p, miR-151-5p, miR-152, miR-154, miR-15a, miR-15b, miR-16, miR-17, miR-181a, miR-181b, miR-181c, miR-185, miR-186, miR-188-5p, miR-18a, miR-18a*, miR-18b, miR-191, miR-192, miR-193a-3p, miR-193a-5p, miR-193b, miR-194, miR-195, miR-196b, miR-197, miR-1979, miR-199a-3p, miR-199b-5p, miR-19a, miR-19b, miR-20a, miR-20b*, miR-21, miR-21*, miR-210, miR-215, miR-217, miR-218, miR-219-5p, miR-22, miR-22*, miR-221, miR-222, miR-23a, miR-23b, miR-24, miR-25, miR-26a, miR-27a, miR-27b, miR-28-5p, miR-296-5p, miR-29a, miR-29b, miR-29c, miR-30b, miR-30c, miR-30c-2*, miR-30d, miR-30e*, miR-32, miR-320a, miR-323-3p, miR-324-3p, miR-324-5p, miR-326, miR-328, miR-337-3p, miR-337-5p, miR-338-3p, miR-339-5p, miR-33b, miR-342-3p, miR-345, miR-346, miR-34a, miR-34c-3p, miR-365, miR-374b, miR-377, miR-378, miR-381, miR-382, miR-409-3p, miR-411, miR-421, miR-423-3p, miR-423-5p, miR-424, miR-425, miR-425*, miR-431, miR-432, miR-433, miR-455-5p, miR-483-3p, miR-484, miR-490-3p, miR-493, miR-497, miR-501-5p, miR-502-5p, miR-503, miR-505, miR-532-5p, miR-574-3p, miR-582-5p, miR-584, miR-589, miR-590-5p, miR-595, miR-597, miR-598, miR-622, miR-629, miR-651, miR-652, miR-660, miR-663, miR-665, miR-671-5p, miR-720, miR-744, miR-877, miR-886-3p, miR-887, miR-888, miR-92a, miR-92b, miR-93, miR-934, miR-940, miR-99a, miR-99b	miR-141, miR-181a*, miR-26a-2*, miR-362-5p, miR-454, miR-514, miR-548b-3p, miR-576-3p, miR-576-5p, miR-654-5p

B

miRNA	Fold	P value
hsa-miR-502-5p	29,50	0,006
hsa-miR-744	23,35	0,032
hsa-miR-373*	19,74	0,010
hsa-miR-572	12,00	0,123
hsa-miR-487b	8,23	0,060
hsa-miR-886-3p	7,80	0,002
hsa-miR-128	7,80	0,013
hsa-miR-431	6,87	0,008
hsa-miR-31*	5,64	0,003
hsa-miR-495	5,03	0,020
hsa-miR-18a*	4,97	0,068
hsa-miR-1979	4,89	0,003
hsa-miR-148a	4,85	0,004
hsa-miR-886-5p	4,75	0,047
hsa-miR-31	4,64	0,005
hsa-miR-154*	4,61	0,017
hsa-miR-720	4,56	0,004
hsa-miR-18a	4,52	0,016
hsa-miR-361-5p	4,35	0,023
hsa-miR-381	4,29	0,174
hsa-miR-223	4,22	0,027
hsa-miR-29b	4,13	0,000
hsa-let-7f	4,13	0,029
hsa-miR-142-3p	3,94	0,038
hsa-let-7d	3,92	0,028
hsa-miR-376a	3,80	0,026
hsa-miR-598	3,69	0,079
hsa-miR-142-5p	3,68	0,009
hsa-miR-410	3,65	0,147
hsa-miR-301a	3,41	0,036
hsa-miR-155	3,36	0,048
hsa-miR-18b	3,35	0,011
hsa-miR-376b	3,33	0,012
hsa-miR-15b	3,20	0,042
hsa-miR-106a	3,14	0,036
hsa-miR-100	3,12	0,002
hsa-miR-766	3,10	0,090
hsa-miR-329	3,02	0,027
hsa-miR-33b	3,01	0,109
hsa-miR-299-5p	2,95	0,165
hsa-miR-33a	2,94	0,006
hsa-miR-17	2,93	0,106
hsa-let-7a	2,92	0,016
hsa-miR-30e*	2,82	0,078
hsa-miR-98	2,80	0,002
hsa-miR-30c	2,76	0,031
hsa-miR-423-3p	2,75	0,068
hsa-miR-411	2,68	0,048
hsa-miR-150	2,64	0,263
hsa-miR-21*	2,60	0,036
hsa-miR-370	2,60	0,032
hsa-miR-30a	2,59	0,052
hsa-miR-151-5p	2,58	0,022
hsa-miR-20a	2,55	0,033
hsa-miR-106b	2,54	0,061
hsa-miR-331-3p	2,50	0,032
hsa-miR-29c	2,50	0,073
hsa-miR-107	2,48	0,004
hsa-miR-127-5p	2,43	0,137
hsa-miR-194	2,42	0,291
hsa-miR-28-5p	2,41	0,023

miRNA	Fold	P value
hsa-miR-339-5p	2,38	0,216
hsa-miR-191	2,36	0,068
hsa-miR-197	2,34	0,023
hsa-miR-140-5p	2,29	0,173
hsa-miR-376c	2,29	0,045
hsa-let-7i	2,29	0,040
hsa-miR-218	2,25	0,294
hsa-miR-127-3p	2,25	0,005
hsa-miR-93	2,19	0,131
hsa-miR-27b	2,17	0,013
hsa-miR-26b	2,17	0,056
hsa-miR-425*	2,17	0,182
hsa-miR-940	2,16	0,023
hsa-miR-337-3p	2,15	0,178
hsa-miR-126*	2,15	0,069
hsa-miR-374a	2,14	0,126
hsa-miR-103	2,12	0,070
hsa-miR-137	2,12	0,030
hsa-miR-425	2,10	0,052
hsa-miR-130b	2,10	0,173
hsa-miR-369-5p	2,09	0,171
hsa-let-7g	2,08	0,105
hsa-miR-24	2,07	0,040
hsa-miR-365	2,04	0,039
hsa-miR-377	1,99	0,096
hsa-miR-23b	1,97	0,057
hsa-miR-30b	1,96	0,065
hsa-miR-126	1,94	0,060
hsa-miR-221	1,93	0,011
hsa-miR-652	1,91	0,025
hsa-let-7c	1,90	0,002
hsa-miR-134	1,90	0,003
hsa-miR-27a	1,87	0,033
hsa-miR-622	1,86	0,030
hsa-miR-20b*	1,84	0,312
hsa-miR-29a	1,80	0,064
hsa-miR-582-5p	1,77	0,292
hsa-miR-181a	1,77	0,019
hsa-miR-30d	1,76	0,150
hsa-miR-16	1,76	0,165
hsa-miR-374b	1,73	0,140
hsa-miR-409-3p	1,71	0,161
hsa-miR-99b	1,71	0,032
hsa-miR-323-3p	1,71	0,092
hsa-miR-214	1,68	0,055
hsa-miR-125a-5p	1,67	0,096
hsa-miR-151-3p	1,67	0,011
hsa-miR-19b	1,63	0,051
hsa-miR-15a	1,61	0,117
hsa-let-7e	1,61	0,030
hsa-miR-186	1,57	0,055
hsa-miR-204	1,57	0,563
hsa-miR-26a	1,57	0,162
hsa-miR-136	1,54	0,003
hsa-miR-602	1,54	0,051
hsa-miR-887	1,54	0,284
hsa-miR-299-3p	1,53	0,469
hsa-miR-125b	1,53	0,244
hsa-miR-509-3p	1,51	0,576
hsa-miR-193a-5p	1,51	0,267
hsa-miR-342-3p	1,50	0,165

miRNA	Fold	P value
hsa-miR-23a	1,49	0,083
hsa-miR-216a	1,47	0,005
hsa-miR-196b	1,47	0,313
hsa-miR-140-3p	1,46	0,515
hsa-miR-338-3p	1,44	0,271
hsa-miR-19a	1,42	0,217
hsa-miR-665	1,42	0,218
hsa-miR-199a-3p	1,41	0,053
hsa-miR-21	1,40	0,054
hsa-miR-382	1,40	0,527
hsa-miR-222	1,37	0,004
hsa-miR-32	1,36	0,142
hsa-let-7d*	1,36	0,314
hsa-let-7b	1,35	0,251
hsa-miR-433	1,34	0,602
hsa-miR-328	1,32	0,521
hsa-miR-660	1,32	0,366
hsa-miR-148b	1,31	0,192
hsa-miR-615-3p	1,30	0,633
hsa-miR-505	1,28	0,197
hsa-miR-132	1,27	0,595
hsa-miR-22*	1,27	0,540
hsa-miR-92a	1,26	0,109
hsa-miR-181b	1,26	0,305
hsa-miR-483-3p	1,25	0,747
hsa-miR-101	1,23	0,429
hsa-miR-326	1,21	0,191
hsa-miR-497	1,20	0,434
hsa-miR-574-3p	1,16	0,486
hsa-miR-345	1,14	0,565
hsa-miR-455-5p	1,12	0,672
hsa-miR-484	1,11	0,432
hsa-miR-185	1,10	0,287
hsa-miR-590-5p	1,09	0,803
hsa-miR-154	1,09	0,821
hsa-miR-217	1,07	0,833
hsa-miR-671-5p	1,06	0,621
hsa-miR-877	1,04	0,957
hsa-miR-486-5p	1,01	0,904
hsa-miR-324-5p	1,01	0,904
hsa-miR-224	1,00	0,986
hsa-miR-25	0,98	0,843
hsa-miR-30c-2*	0,95	0,942
hsa-miR-22	0,94	0,708
hsa-miR-188-5p	0,93	0,475
hsa-miR-490-3p	0,92	0,867
hsa-miR-324-3p	0,92	0,791
hsa-miR-378	0,91	0,634
hsa-miR-452	0,90	0,888
hsa-miR-532-5p	0,89	0,791
hsa-miR-663	0,84	0,414
hsa-miR-337-5p	0,84	0,687
hsa-miR-10b	0,83	0,287
hsa-miR-361-3p	0,78	0,580
hsa-miR-199a-5p	0,76	0,729
hsa-miR-99a	0,76	0,103
hsa-miR-215	0,76	0,560
hsa-miR-144	0,75	0,308
hsa-miR-346	0,74	0,473
hsa-miR-192	0,72	0,363
hsa-miR-320a	0,71	0,075

miRNA	Fold	P value
hsa-miR-589	0,67	0,234
hsa-miR-500a	0,66	0,273
hsa-miR-888	0,65	0,373
hsa-miR-193b	0,64	0,072
hsa-miR-130a	0,64	0,076
hsa-miR-934	0,61	0,026
hsa-miR-152	0,60	0,027
hsa-miR-595	0,60	0,447
hsa-miR-423-5p	0,59	0,095
hsa-miR-451	0,55	0,122
hsa-miR-450a	0,49	0,262
hsa-miR-34c-3p	0,44	0,043
hsa-miR-34a	0,41	0,015
hsa-miR-181c	0,38	0,077
hsa-miR-210	0,36	0,122
hsa-miR-424	0,34	0,011
hsa-miR-193a-3p	0,34	0,010
hsa-miR-542-5p	0,25	0,006
hsa-miR-503	0,17	0,089
hsa-miR-205	0,15	0,037
hsa-miR-146a	0,13	0,005

Figure S2

(A) Table of miRNA common between HUVECs and exosomes or only detected in exosomes or in producing HUVECs. (B) Table of the 204 miRNAs detected in exosomes from HUVECs cultured in basal or tumoral displaying a variation lower than 2 between replicates and an individual Ct value lower than 40.

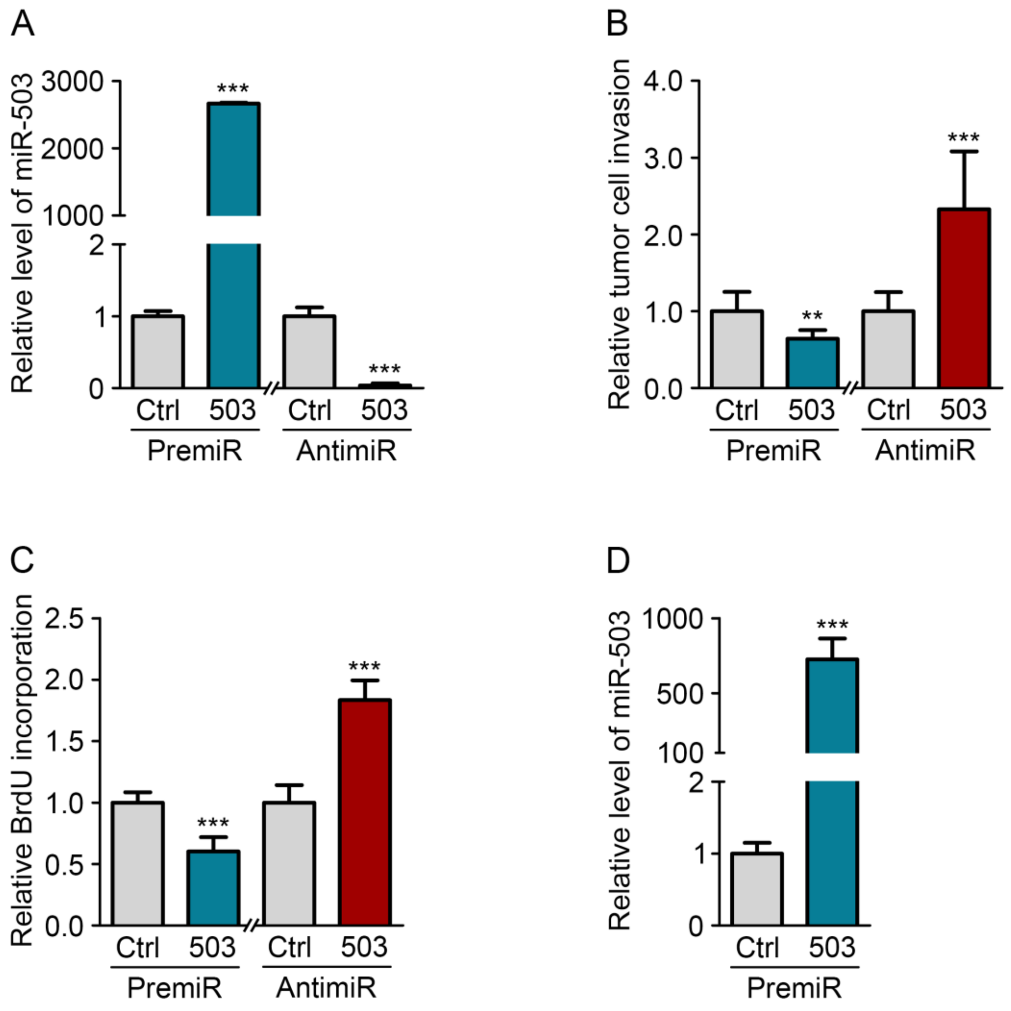

Figure S3

(A) MiR-503 levels, evaluated by qRT-PCR, in MDA-MB-231 cells transfected with pre-miR-control or pre-miR-503 and with anti-miR-control or anti-miR-503. (B) Boyden invasion assay of MDA-MB-231 cells transfected with pre-miR-control or pre-miR-503 and with anti-miR-control or anti-miR-503. (C) BrdU incorporation in MDA-MB-231 cells transfected with pre-miR-control or pre-miR-503 and with anti-miR-control or anti-miR-503. (D) MiR-503 levels, evaluated by qRT-PCR, in HUVECs transfected with pre-miR-control or pre-miR-503.

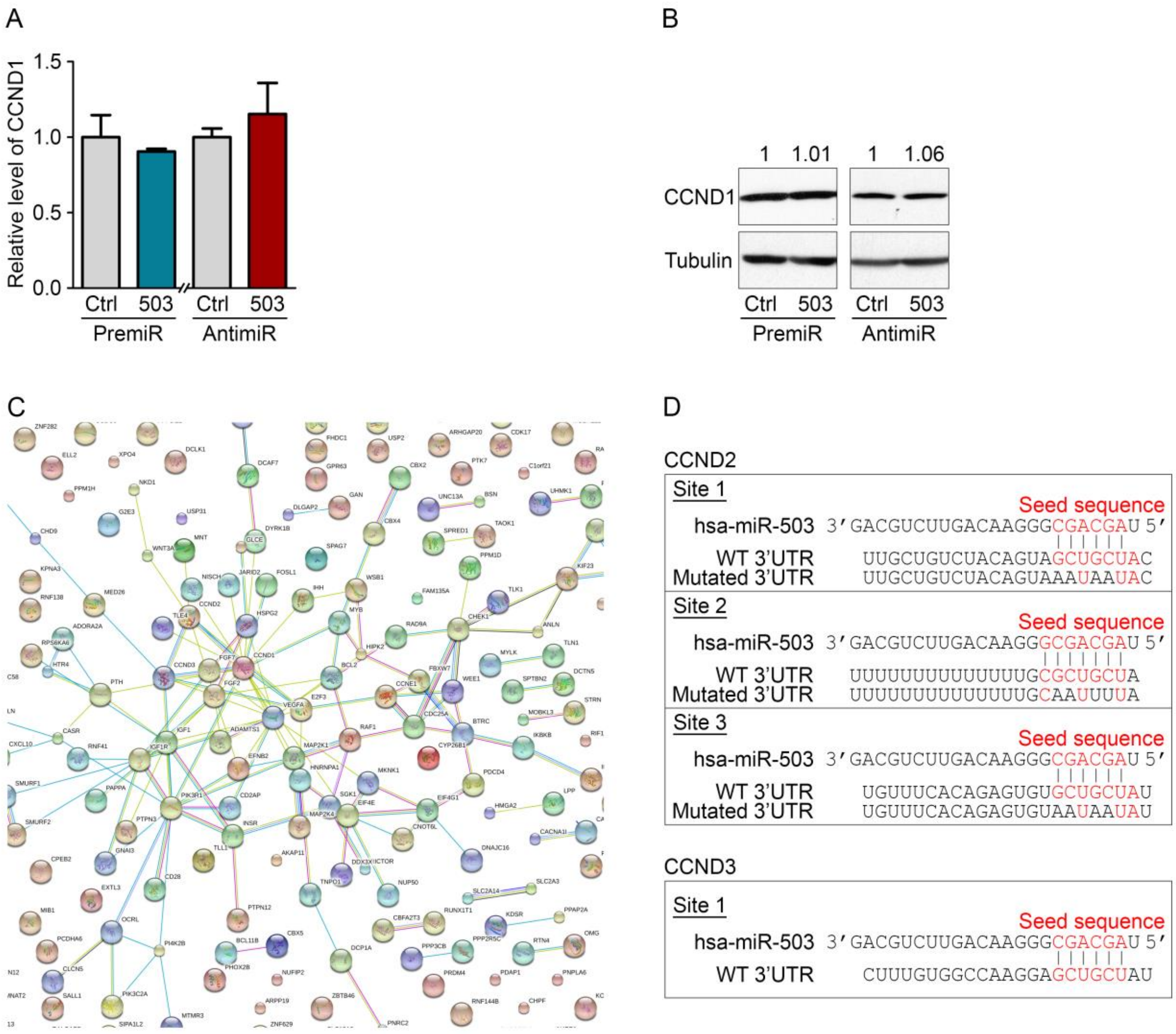

Figure S4

(A) mRNA levels, quantified by qRT-PCR, of CCND1 in MDA-MB-231 cells transfected with pre-miR-503 or pre-miR-control and with anti-miR-control or anti–miR-503 and starved for 36 h. (B) Western blotting of CCND1 in MDA-MB-231 cells transfected with pre-miR-503 or pre-miR-control and with anti-miR-control or anti-miR-503 and starved for 36 h. (C) Bioinformatics analysis of miR-503 target genes predicted by Targetscan using String 9.1 software. (D) Predicted miR-503 binding sites within the 3'-UTRs of CCND2 and CCND3.
